# Crystal structure of 7-phenyl-7-(2,4,5-trimethyl-3,6-dioxo­cyclo­hexa-1,4-dien-1-yl)hepta­noate 1,3-dihy­droxy-2-(hy­droxy­meth­yl)propan-2-aminium monohydrate: a new solid form of seratrodast

**DOI:** 10.1107/S1600536814020625

**Published:** 2014-09-20

**Authors:** Benyong Lou

**Affiliations:** a Department of Chemistry and Chemical Engineering, Minjiang University, Fuzhou, 350108, People’s Republic of China, Corresponding E-mail: lby@mju.edu.cn

**Keywords:** crystal structure, seratrodast, trometamol, salt, solubility, hydrogen bonding

## Abstract

In the title hydrated salt, C_4_H_12_NO_3_^+^·C_22_H_25_O_4_^−^·H_2_O, seratrodast [systematic name: 7-phenyl-7-(2,4,5-trimethyl-3,6-dioxo­cyclo­hexa-1,4-dien-1-yl)hepta­noic acid] crystallized with trometamol [systematic name: 2-amino-2-(hydroxyméth­yl)propane-1,3-diol] to form a monohydrated salt form of seratrodast. The carb­oxy­lic acid group of seratrodast has transferred its proton to the amino N atom of trometamol. In the crystal, the trometamol cations are linked to the water mol­ecules and to each other by N—H⋯O and O—H⋯O hydrogen bonds forming sheets parallel to (100). The seratrodast anions are linked to both sides of these sheets by O—H⋯O and C—H⋯O hydrogen bonds, forming a three-layer two-dimensional structure. After forming the title salt, the solubility of seratrodast was found to be greatly improved.

## Abstract 2

In the title compound, seratrodast has crystallized with trometamol to form a monohydrated salt. The carb­oxy­lic acid group of seratrodast has transferred its proton to the amino N atom of trometamol.

## Chemical context   

Seratrodast is the first thromboxane A2 receptor antagonist to have been developed as an anti-asthmatic drug (Samara, 1996 ▶). This drug mol­ecule with a carb­oxy­lic group is practically insoluble in water. Its new solid forms have been scarcely exploited and only a polymorphic transition was ever investigated (Urakami & Beezer, 2003 ▶). Tris(hy­droxy­meth­yl)amino methane, commonly called trometamol, is often used as a buffer in biochemical studies. It has been successfully exploited for improving properties of APIs such as ketoprofen (Zippel & Wagenitz, 2006 ▶). In this study, trometamol was employed to co-crystallize with seratrodast to give rise to a hydrated salt. To the best of our knowledge, the title salt is the first multi-component crystalline form of seratrodast to be reported.
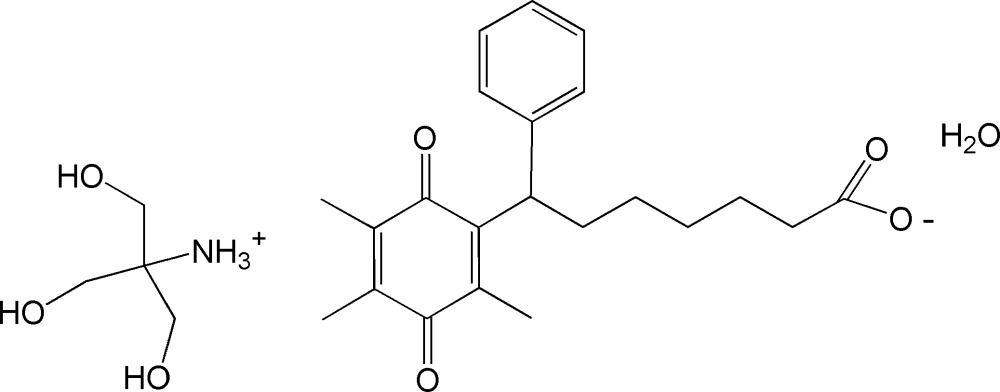


## Structural commentary   

**Figure 1:**
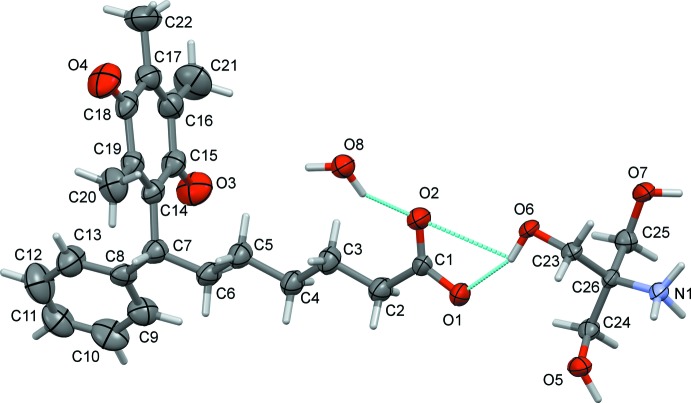
A view of the mol­ecular structure of the title salt, with atom labelling. Displacement ellipsoids are drawn at the 50% probability level. Hydrogen bonds are shown as dashed lines (see Table 1 ▶ for details).

The mol­ecular structure of the title salt is illustrated in Fig. 1 ▶. It was clear from a difference Fourier map that the carb­oxy­lic group of seratrodast had transferred its proton to the amino N atom of trometamol. The bond distances C1—O1 and C1—O2 of the carboxyl­ate group of the seratrodast anion are 1.258 (4) and 1.232 (4) Å, respectively. The phenyl ring is normal to the dioxo­cyclo­hexa­diene ring, with a dihedral angle of 89.95 (19)°, and the alkyl chain has an extended conformation.

## Supra­molecular features   

**Figure 2:**
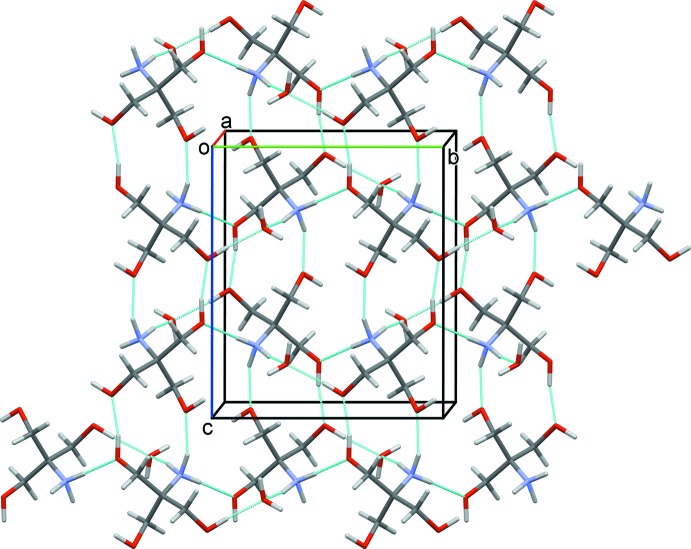
A view along the *a* axis of the two-dimensional hydrogen-bonded structure of the trometamol cations and the water mol­ecules (hydrogen bonds are shown as dashed lines; see Table 1 ▶ for details).

**Figure 3:**
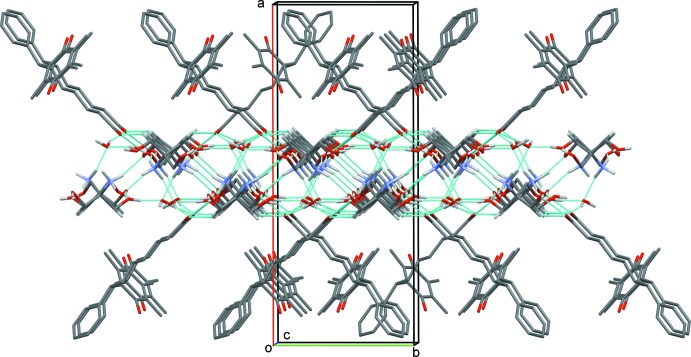
A view along the *c* axis of the crystal packing of the title compound (hydrogen bonds are shown as dashed lines; see Table 1 ▶ for details). H atoms not involved in hydrogen bonding have been omitted for clarity

In the crystal, the trometamol cations are linked to the water mol­ecules and to each other by N—H⋯O and O—H⋯O hydrogen bonds, forming sheets parallel to (100); see Table 1 ▶ and Fig. 2 ▶. The seratrodast anions are linked to both sides of these sheets by O—H⋯O and C—H⋯O hydrogen bonds, forming a three-layer two-dimensional structure (Fig. 3 ▶ and Table 1 ▶). Further details of the hydrogen bonding are given below and in Table 1 ▶. The carboxyl­ate anion inter­acts with one hydroxyl group of trometamol through strong hydrogen bonding [O6⋯O1 = 2.662 (3) Å]. There also exist hydrogen-bonding inter­actions between carboxyl­ate anion and water mol­ecule [O8⋯O2 = 2.617 (3) Å, O8⋯O1^i^ = 2.667 (3) Å]. The protonated trometamol cation inter­acts with each other through three kinds of hydrogen-bonding inter­actions. An 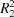
(11) heterosynthon is formed through hydrogen-bonding inter­actions between the hydroxyl groups [O5⋯O7^iii^ = 2.714 (3) Å] and between the hydroxyl group and the amino group [N1⋯O6^iii^ = 2.779 (3) Å]. Along the *c* axis, the 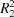
(11) heterosynthon gives rise to a hydrogen-bonded chain of trometamol cations, which is further linked into a two-dimensional structure by hydrogen-bonding inter­actions between the amino and the hydroxyl groups [N1⋯O5^ii^ = 2.935 (3) Å]. There also exist hydrogen-bonding inter­actions between water and trometamol [N1⋯O8^i^ = 2.800 (4) Å; O7⋯O8^iii^ = 2.686 (3) Å]. The various hydrogen-bonding inter­actions result in a two-dimensional layer structure in which the seratrodast anions are spread around two sides of the layer in an orderly manner (Table 1 ▶ and Fig. 3 ▶).

## Database survey   

To the best of our knowledge, the title salt is the first multi-component crystalline form of seratrodast to be reported.

## Synthesis and crystallization   

Seratrodast (354 mg, 1 mmol) and trometamol (121 mg, 1 mmol) were dissolved in methanol (15 ml). The resulting solution was kept in air and after several days yellow block-like crystals of the title salt were obtained.

## Solubility Studies   

Excess amounts of seratrodast and the title salt were suspended in 10 ml of water in screw-capped glass vials, respectively. These vials were kept at 310 K and were stirred at 100 r.p.m. using a magnetic stirrer. After 72 h, the suspensions were filtered through a 0.2 µm syringe filter. The filtered aliquots were sufficiently diluted, and the absorbances were measured at 268 nm in triplicate. Finally, the concentration of seratrodast after 72 h in each sample was determined from the previously made standard graph. A standard graph was made by measuring the absorbance of varied concentrations of seratrodast (2–16 mg/*L*) in water/methanol (9:1) solution using a UV-2500 spectrophotometer at 268 nm. The calibrated plot showed a good correlation coefficient (*y* = 0.04997*x* + 0.00459, *R* ^2^ = 0.9991). After forming the title salt, the solubility of seratrodast was found to be greatly improved.

## Refinement   

Crystal data, data collection and structure refinement details are summarized in Table 2 ▶. The C-bound H atoms were positioned geometrically and refined as riding atoms: C—H = 0.95–1.00 Å with *U*_iso_(H) = 1.2*U*_eq_(C). The OH and NH_3_^+^ H atoms were located in difference Fourier maps and refined as riding atoms with *U*_iso_(H) = 1.2*U*_eq_(O,*N*).

## Supplementary Material

Crystal structure: contains datablock(s) I, lou. DOI: 10.1107/S1600536814020625/su2780sup1.cif

Structure factors: contains datablock(s) I. DOI: 10.1107/S1600536814020625/su2780Isup2.hkl

Supporting information file. DOI: 10.1107/S1600536814020625/su2780Isup3.cml

CCDC reference: 975488

Additional supporting information:  crystallographic information; 3D view; checkCIF report

## Figures and Tables

**Table 1:** Hydrogen-bond geometry (Å, °)

*D*—H⋯*A*	*D*—H	H⋯*A*	*D*⋯*A*	*D*—H⋯*A*
N1—H1*C*⋯O8^i^	0.99	1.85	2.800 (4)	160
N1—H1*B*⋯O5^ii^	0.91	2.04	2.935 (3)	166
N1—H1*A*⋯O6^iii^	0.97	1.88	2.779 (3)	153
O5—H5⋯O7^iii^	0.97	1.76	2.714 (3)	170
O6—H6⋯O1	0.91	1.81	2.662 (3)	154
O7—H7*A*⋯O8^iii^	0.92	1.77	2.686 (3)	173
O8—H8*B*⋯O2	0.92	1.76	2.617 (3)	1523
O8—H8*A*⋯O1^iv^	0.91	1.76	2.667 (3)	173
C24—H24*A*⋯O1	0.99	2.55	3.410 (4)	146
C25—H25*A*⋯O2^iii^	0.99	2.44	3.326 (4)	149

Symmetry codes: (i) 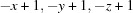; (ii) 
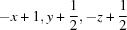
; (iii) 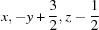; (iv) 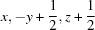.

**Table 2:** Experimental details

Crystal data	
Chemical formula	C_4_H_12_NO_3_^+^·C_22_H_25_O_4_^−^·H_2_O
*M*_r_	493.58
Crystal system, space group	Monoclinic, *P*2_1_/*c*
Temperature (K)	293
*a*, *b*, *c* (Å)	23.506 (9), 9.665 (4), 11.344 (5)
β (°)	94.223 (7)
*V* (Å^3^)	2570.0 (17)
*Z*	4
Radiation type	Mo *K*α
μ (mm^−1^)	0.09
Crystal size (mm)	0.20 × 0.20 × 0.20

Data collection	
Diffractometer	Rigaku Mercury CCD
Absorption correction	Multi-scan (*CrystalClear*; Rigaku, 2000 ▶)
*T*_min_, *T*_max_	0.549, 1.000
No. of measured, independent and observed [*I* > 2σ(*I*)] reflections	20028, 5762, 3564
*R*_int_	0.062
(sin θ/λ)_max_ (Å^−1^)	0.649

Refinement	
*R*[*F* ^2^ > 2σ(*F* ^2^)], *wR*(*F* ^2^), *S*	0.074, 0.288, 1.10
No. of reflections	5762
No. of parameters	319
H-atom treatment	H-atom parameters constrained
Δρ_max_, Δρ_min_ (e Å^−3^)	0.55, −0.42

Computer programs: *CrystalClear* (Rigaku, 2000 ▶), *SHELXS97* and *SHELXL97* (Sheldrick, 2008 ▶), *Mercury* (Macrae *et al.*, 2008 ▶), *PLATON* (Spek, 2009 ▶) and *publCIF* (Westrip, 2010 ▶).

## supplementary crystallographic information

### Crystal data

**Table:**

C_4_H_12_NO_3_^+^·C_22_H_25_O_4_^−^·H_2_O	*F*(000) = 1064
*M**_r_* = 493.58	*D*_x_ = 1.276 Mg m^−^^3^
Monoclinic, *P*2_1_/*c*	Mo *K*α radiation, λ = 0.71073 Å
Hall symbol: -P 2ybc	Cell parameters from 6295 reflections
*a* = 23.506 (9) Å	θ = 2.1–27.5°
*b* = 9.665 (4) Å	µ = 0.09 mm^−^^1^
*c* = 11.344 (5) Å	*T* = 293 K
β = 94.223 (7)°	Prism, yellow
*V* = 2570.0 (17) Å^3^	0.20 × 0.20 × 0.20 mm
*Z* = 4	

### Data collection

**Table:**

Rigaku Mercury CCD diffractometer	5762 independent reflections
Radiation source: fine-focus sealed tube	3564 reflections with *I* > 2σ(*I*)
Graphite monochromator	*R*_int_ = 0.062
Detector resolution: 28.5714 pixels mm^-1^	θ_max_ = 27.5°, θ_min_ = 2.6°
CCD_Profile_fitting scans	*h* = −30→30
Absorption correction: multi-scan (*CrystalClear*; Rigaku, 2000)	*k* = −12→12
*T*_min_ = 0.549, *T*_max_ = 1.000	*l* = −12→14
20028 measured reflections	

### Refinement

**Table:**

Refinement on *F*^2^	Primary atom site location: structure-invariant direct methods
Least-squares matrix: full	Secondary atom site location: difference Fourier map
*R*[*F*^2^ > 2σ(*F*^2^)] = 0.074	Hydrogen site location: inferred from neighbouring sites
*wR*(*F*^2^) = 0.288	H-atom parameters constrained
*S* = 1.10	*w* = 1/[σ^2^(*F*_o_^2^) + (0.1567*P*)^2^] where *P* = (*F*_o_^2^ + 2*F*_c_^2^)/3
5762 reflections	(Δ/σ)_max_ < 0.001
319 parameters	Δρ_max_ = 0.55 e Å^−^^3^
0 restraints	Δρ_min_ = −0.42 e Å^−^^3^

### Special details

**Table:**

Geometry. All e.s.d.'s (except the e.s.d. in the dihedral angle between two l.s. planes) are estimated using the full covariance matrix. The cell e.s.d.'s are taken into account individually in the estimation of e.s.d.'s in distances, angles and torsion angles; correlations between e.s.d.'s in cell parameters are only used when they are defined by crystal symmetry. An approximate (isotropic) treatment of cell e.s.d.'s is used for estimating e.s.d.'s involving l.s. planes.
Refinement. Refinement of *F*^2^ against ALL reflections. The weighted *R*-factor *wR* and goodness of fit *S* are based on *F*^2^, conventional *R*-factors *R* are based on *F*, with *F* set to zero for negative *F*^2^. The threshold expression of *F*^2^ > σ(*F*^2^) is used only for calculating *R*-factors(gt) *etc*. and is not relevant to the choice of reflections for refinement. *R*-factors based on *F*^2^ are statistically about twice as large as those based on *F*, and *R*- factors based on ALL data will be even larger.

### Fractional atomic coordinates and isotropic or equivalent isotropic displacement parameters (Å^2^)

**Table:**

	*x*	*y*	*z*	*U*_iso_*/*U*_eq_	
O1	0.38025 (10)	0.4145 (2)	0.4397 (2)	0.0447 (6)	
O2	0.38047 (12)	0.4204 (3)	0.6328 (2)	0.0537 (7)	
O3	0.09684 (12)	0.0531 (3)	0.8988 (3)	0.0749 (9)	
O4	0.25430 (13)	−0.0713 (4)	1.2339 (3)	0.0789 (10)	
O5	0.44774 (10)	0.5677 (2)	0.17616 (18)	0.0408 (6)	
O6	0.44283 (9)	0.6368 (2)	0.49902 (17)	0.0389 (6)	
O7	0.42596 (11)	0.9565 (2)	0.43874 (19)	0.0482 (6)	
O8	0.42283 (9)	0.2989 (2)	0.8260 (2)	0.0440 (6)	
H8B	0.4079	0.3136	0.7498	0.053*	
H1C	0.5077	0.7821	0.1992	0.053*	
H7A	0.4222	1.0380	0.3969	0.053*	
H1B	0.4962	0.9171	0.2650	0.053*	
H1A	0.4590	0.8684	0.1637	0.053*	
H8A	0.4059	0.2252	0.8599	0.053*	
H5	0.4389	0.5703	0.0917	0.053*	
H6	0.4274	0.5623	0.4592	0.053*	
N1	0.47892 (10)	0.8382 (3)	0.2369 (2)	0.0333 (6)	
C1	0.36324 (13)	0.3727 (3)	0.5360 (3)	0.0339 (7)	
C2	0.31629 (15)	0.2633 (4)	0.5285 (3)	0.0469 (8)	
H2A	0.2801	0.3087	0.5001	0.056*	
H2B	0.3254	0.1944	0.4681	0.056*	
C3	0.30659 (14)	0.1871 (4)	0.6411 (3)	0.0470 (8)	
H3A	0.3018	0.2554	0.7047	0.056*	
H3B	0.3408	0.1309	0.6644	0.056*	
C4	0.25457 (15)	0.0926 (4)	0.6304 (3)	0.0465 (8)	
H4A	0.2204	0.1495	0.6080	0.056*	
H4B	0.2592	0.0259	0.5656	0.056*	
C5	0.24405 (15)	0.0123 (4)	0.7421 (3)	0.0502 (9)	
H5A	0.2750	−0.0560	0.7580	0.060*	
H5B	0.2448	0.0769	0.8099	0.060*	
C6	0.18728 (15)	−0.0623 (4)	0.7312 (3)	0.0475 (9)	
H6A	0.1567	0.0078	0.7184	0.057*	
H6B	0.1861	−0.1212	0.6597	0.057*	
C7	0.17374 (14)	−0.1507 (4)	0.8334 (3)	0.0452 (8)	
H7	0.2046	−0.2222	0.8397	0.054*	
C8	0.11810 (13)	−0.2336 (4)	0.8148 (3)	0.0412 (8)	
C9	0.08118 (15)	−0.2237 (4)	0.7166 (4)	0.0566 (10)	
H9	0.0876	−0.1562	0.6581	0.068*	
C10	0.03397 (16)	−0.3118 (5)	0.7012 (5)	0.0725 (13)	
H10	0.0092	−0.3051	0.6314	0.087*	
C13	0.10716 (18)	−0.3320 (5)	0.8987 (4)	0.0670 (12)	
H13	0.1327	−0.3426	0.9670	0.080*	
C12	0.0590 (2)	−0.4157 (5)	0.8836 (5)	0.0860 (16)	
H12	0.0512	−0.4800	0.9437	0.103*	
C11	0.02316 (17)	−0.4075 (5)	0.7856 (5)	0.0689 (12)	
H11	−0.0090	−0.4671	0.7752	0.083*	
C14	0.17815 (13)	−0.0778 (3)	0.9519 (3)	0.0397 (8)	
C15	0.13375 (14)	0.0248 (4)	0.9771 (3)	0.0463 (9)	
C16	0.13441 (16)	0.0942 (4)	1.0924 (4)	0.0531 (9)	
C19	0.22057 (13)	−0.1042 (4)	1.0361 (3)	0.0426 (8)	
C18	0.21906 (15)	−0.0386 (4)	1.1559 (3)	0.0494 (9)	
C17	0.17570 (17)	0.0652 (4)	1.1788 (4)	0.0555 (10)	
C20	0.27017 (17)	−0.1961 (4)	1.0232 (4)	0.0655 (11)	
H20A	0.2645	−0.2481	0.9491	0.098*	
H20B	0.2740	−0.2607	1.0898	0.098*	
H20C	0.3049	−0.1401	1.0220	0.098*	
C21	0.0871 (2)	0.1955 (6)	1.1065 (5)	0.0896 (16)	
H21A	0.0928	0.2408	1.1838	0.134*	
H21B	0.0505	0.1466	1.1011	0.134*	
H21C	0.0871	0.2653	1.0439	0.134*	
C22	0.1798 (2)	0.1297 (6)	1.2988 (4)	0.0891 (17)	
H22A	0.2194	0.1556	1.3207	0.134*	
H22B	0.1669	0.0634	1.3565	0.134*	
H22C	0.1556	0.2125	1.2979	0.134*	
C23	0.47623 (12)	0.6918 (3)	0.4125 (2)	0.0319 (6)	
H23A	0.4992	0.6168	0.3801	0.038*	
H23B	0.5029	0.7612	0.4494	0.038*	
C24	0.40803 (12)	0.6541 (3)	0.2309 (3)	0.0346 (7)	
H24A	0.3833	0.5965	0.2781	0.042*	
H24B	0.3834	0.7023	0.1693	0.042*	
C25	0.39738 (13)	0.8612 (3)	0.3592 (3)	0.0392 (7)	
H25A	0.3773	0.9124	0.2930	0.047*	
H25B	0.3686	0.8093	0.4008	0.047*	
C26	0.43939 (11)	0.7603 (3)	0.3108 (2)	0.0296 (6)	

### Atomic displacement parameters (Å^2^)

**Table:**

	*U*^11^	*U*^22^	*U*^33^	*U*^12^	*U*^13^	*U*^23^
O1	0.0627 (15)	0.0412 (12)	0.0308 (13)	−0.0161 (10)	0.0075 (10)	0.0027 (10)
O2	0.0810 (18)	0.0487 (14)	0.0311 (13)	−0.0191 (12)	0.0032 (12)	−0.0005 (11)
O3	0.0648 (18)	0.090 (2)	0.067 (2)	0.0198 (16)	−0.0195 (15)	0.0060 (17)
O4	0.0712 (19)	0.099 (3)	0.062 (2)	−0.0058 (17)	−0.0235 (15)	0.0159 (18)
O5	0.0564 (13)	0.0365 (11)	0.0280 (12)	0.0050 (10)	−0.0064 (10)	−0.0036 (9)
O6	0.0626 (14)	0.0380 (12)	0.0156 (10)	−0.0120 (10)	−0.0012 (9)	0.0025 (9)
O7	0.0854 (18)	0.0351 (12)	0.0226 (12)	0.0038 (11)	−0.0057 (11)	0.0000 (9)
O8	0.0505 (13)	0.0447 (13)	0.0368 (13)	−0.0080 (10)	0.0037 (10)	−0.0006 (10)
N1	0.0433 (14)	0.0333 (12)	0.0227 (13)	−0.0094 (10)	−0.0014 (10)	0.0024 (10)
C1	0.0450 (17)	0.0294 (14)	0.0279 (17)	0.0010 (12)	0.0072 (13)	0.0024 (12)
C2	0.052 (2)	0.053 (2)	0.0360 (19)	−0.0131 (16)	0.0066 (15)	0.0017 (16)
C3	0.0459 (18)	0.052 (2)	0.044 (2)	−0.0116 (15)	0.0043 (15)	0.0099 (16)
C4	0.0498 (19)	0.052 (2)	0.038 (2)	−0.0124 (15)	0.0065 (15)	0.0046 (16)
C5	0.053 (2)	0.057 (2)	0.040 (2)	−0.0163 (16)	0.0010 (16)	0.0065 (17)
C6	0.052 (2)	0.056 (2)	0.0357 (19)	−0.0158 (16)	0.0109 (15)	−0.0008 (16)
C7	0.0446 (18)	0.053 (2)	0.0377 (19)	−0.0103 (15)	0.0033 (14)	−0.0009 (16)
C8	0.0364 (16)	0.0511 (19)	0.0372 (18)	−0.0077 (14)	0.0110 (13)	−0.0085 (15)
C9	0.045 (2)	0.066 (2)	0.059 (2)	−0.0098 (17)	0.0019 (17)	−0.006 (2)
C10	0.043 (2)	0.083 (3)	0.089 (3)	−0.010 (2)	−0.015 (2)	−0.017 (3)
C13	0.061 (2)	0.081 (3)	0.059 (3)	−0.031 (2)	0.0041 (19)	0.004 (2)
C12	0.067 (3)	0.073 (3)	0.119 (5)	−0.032 (2)	0.011 (3)	0.008 (3)
C11	0.044 (2)	0.064 (3)	0.099 (4)	−0.0171 (19)	0.003 (2)	−0.012 (3)
C14	0.0358 (16)	0.0472 (18)	0.0365 (18)	−0.0106 (13)	0.0043 (13)	0.0026 (15)
C15	0.0420 (18)	0.053 (2)	0.043 (2)	−0.0035 (15)	−0.0028 (15)	0.0080 (16)
C16	0.056 (2)	0.048 (2)	0.057 (2)	−0.0016 (16)	0.0137 (18)	−0.0014 (18)
C19	0.0399 (17)	0.0440 (17)	0.044 (2)	−0.0069 (13)	0.0005 (14)	0.0041 (15)
C18	0.0477 (19)	0.055 (2)	0.044 (2)	−0.0183 (16)	−0.0044 (16)	0.0112 (17)
C17	0.064 (2)	0.054 (2)	0.049 (2)	−0.0185 (18)	0.0087 (18)	−0.0054 (18)
C20	0.053 (2)	0.060 (2)	0.082 (3)	0.0060 (18)	−0.003 (2)	0.009 (2)
C21	0.087 (3)	0.082 (4)	0.101 (4)	0.029 (3)	0.021 (3)	−0.009 (3)
C22	0.120 (4)	0.092 (4)	0.058 (3)	−0.029 (3)	0.025 (3)	−0.026 (3)
C23	0.0401 (15)	0.0323 (14)	0.0224 (15)	−0.0022 (12)	−0.0033 (12)	0.0006 (12)
C24	0.0361 (15)	0.0371 (15)	0.0297 (16)	−0.0072 (12)	−0.0043 (12)	0.0038 (13)
C25	0.0427 (17)	0.0362 (16)	0.0385 (18)	0.0011 (13)	0.0009 (13)	0.0006 (14)
C26	0.0362 (15)	0.0316 (14)	0.0210 (14)	−0.0061 (11)	0.0009 (11)	0.0043 (12)

### Geometric parameters (Å, º)

**Table:**

O1—C1	1.258 (4)	C9—C10	1.400 (5)
O2—C1	1.232 (4)	C9—H9	0.9500
O3—C15	1.226 (4)	C10—C11	1.369 (7)
O4—C18	1.209 (4)	C10—H10	0.9500
O5—C24	1.428 (4)	C13—C12	1.392 (5)
O5—H5	0.9657	C13—H13	0.9500
O6—C23	1.406 (4)	C12—C11	1.347 (7)
O6—H6	0.9109	C12—H12	0.9500
O7—C25	1.423 (4)	C11—H11	0.9500
O7—H7A	0.9202	C14—C19	1.353 (4)
O8—H8B	0.9191	C14—C15	1.483 (5)
O8—H8A	0.9142	C15—C16	1.469 (5)
N1—C26	1.499 (4)	C16—C17	1.357 (6)
N1—H1C	0.9892	C16—C21	1.499 (6)
N1—H1B	0.9100	C19—C20	1.482 (5)
N1—H1A	0.9682	C19—C18	1.502 (5)
C1—C2	1.526 (4)	C18—C17	1.467 (6)
C2—C3	1.507 (5)	C17—C22	1.493 (6)
C2—H2A	0.9900	C20—H20A	0.9800
C2—H2B	0.9900	C20—H20B	0.9800
C3—C4	1.523 (4)	C20—H20C	0.9800
C3—H3A	0.9900	C21—H21A	0.9800
C3—H3B	0.9900	C21—H21B	0.9800
C4—C5	1.522 (5)	C21—H21C	0.9800
C4—H4A	0.9900	C22—H22A	0.9800
C4—H4B	0.9900	C22—H22B	0.9800
C5—C6	1.514 (5)	C22—H22C	0.9800
C5—H5A	0.9900	C23—C26	1.540 (4)
C5—H5B	0.9900	C23—H23A	0.9900
C6—C7	1.493 (5)	C23—H23B	0.9900
C6—H6A	0.9900	C24—C26	1.524 (4)
C6—H6B	0.9900	C24—H24A	0.9900
C7—C14	1.515 (5)	C24—H24B	0.9900
C7—C8	1.535 (4)	C25—C26	1.519 (4)
C7—H7	1.0000	C25—H25A	0.9900
C8—C9	1.364 (5)	C25—H25B	0.9900
C8—C13	1.383 (5)		
			
C24—O5—H5	108.4	C12—C11—C10	118.7 (4)
C23—O6—H6	100.0	C12—C11—H11	120.6
C25—O7—H7A	101.8	C10—C11—H11	120.6
H8B—O8—H8A	111.6	C19—C14—C15	118.8 (3)
C26—N1—H1C	116.1	C19—C14—C7	122.5 (3)
C26—N1—H1B	120.2	C15—C14—C7	118.7 (3)
H1C—N1—H1B	108.0	O3—C15—C16	120.2 (4)
C26—N1—H1A	110.6	O3—C15—C14	118.7 (3)
H1C—N1—H1A	95.6	C16—C15—C14	121.1 (3)
H1B—N1—H1A	102.8	C17—C16—C15	120.6 (4)
O2—C1—O1	123.3 (3)	C17—C16—C21	123.7 (4)
O2—C1—C2	119.9 (3)	C15—C16—C21	115.7 (4)
O1—C1—C2	116.7 (3)	C14—C19—C20	126.0 (3)
C3—C2—C1	116.4 (3)	C14—C19—C18	119.6 (3)
C3—C2—H2A	108.2	C20—C19—C18	114.4 (3)
C1—C2—H2A	108.2	O4—C18—C17	119.8 (4)
C3—C2—H2B	108.2	O4—C18—C19	119.4 (4)
C1—C2—H2B	108.2	C17—C18—C19	120.8 (3)
H2A—C2—H2B	107.3	C16—C17—C18	118.8 (4)
C2—C3—C4	113.3 (3)	C16—C17—C22	124.5 (4)
C2—C3—H3A	108.9	C18—C17—C22	116.7 (4)
C4—C3—H3A	108.9	C19—C20—H20A	109.5
C2—C3—H3B	108.9	C19—C20—H20B	109.5
C4—C3—H3B	108.9	H20A—C20—H20B	109.5
H3A—C3—H3B	107.7	C19—C20—H20C	109.5
C5—C4—C3	114.7 (3)	H20A—C20—H20C	109.5
C5—C4—H4A	108.6	H20B—C20—H20C	109.5
C3—C4—H4A	108.6	C16—C21—H21A	109.5
C5—C4—H4B	108.6	C16—C21—H21B	109.5
C3—C4—H4B	108.6	H21A—C21—H21B	109.5
H4A—C4—H4B	107.6	C16—C21—H21C	109.5
C6—C5—C4	111.8 (3)	H21A—C21—H21C	109.5
C6—C5—H5A	109.3	H21B—C21—H21C	109.5
C4—C5—H5A	109.3	C17—C22—H22A	109.5
C6—C5—H5B	109.3	C17—C22—H22B	109.5
C4—C5—H5B	109.3	H22A—C22—H22B	109.5
H5A—C5—H5B	107.9	C17—C22—H22C	109.5
C7—C6—C5	116.5 (3)	H22A—C22—H22C	109.5
C7—C6—H6A	108.2	H22B—C22—H22C	109.5
C5—C6—H6A	108.2	O6—C23—C26	111.9 (2)
C7—C6—H6B	108.2	O6—C23—H23A	109.2
C5—C6—H6B	108.2	C26—C23—H23A	109.2
H6A—C6—H6B	107.3	O6—C23—H23B	109.2
C6—C7—C14	114.7 (3)	C26—C23—H23B	109.2
C6—C7—C8	114.9 (3)	H23A—C23—H23B	107.9
C14—C7—C8	111.5 (3)	O5—C24—C26	110.5 (2)
C6—C7—H7	104.8	O5—C24—H24A	109.6
C14—C7—H7	104.8	C26—C24—H24A	109.6
C8—C7—H7	104.8	O5—C24—H24B	109.6
C9—C8—C13	118.1 (3)	C26—C24—H24B	109.6
C9—C8—C7	124.0 (3)	H24A—C24—H24B	108.1
C13—C8—C7	117.7 (3)	O7—C25—C26	110.8 (2)
C8—C9—C10	120.7 (4)	O7—C25—H25A	109.5
C8—C9—H9	119.7	C26—C25—H25A	109.5
C10—C9—H9	119.7	O7—C25—H25B	109.5
C11—C10—C9	120.7 (4)	C26—C25—H25B	109.5
C13—C10—H10	119.7	H25A—C25—H25B	108.1
C9—C10—H10	119.7	N1—C26—C25	109.1 (2)
C8—C13—C12	120.4 (4)	N1—C26—C24	107.3 (2)
C8—C13—H13	119.8	C25—C26—C24	110.4 (2)
C12—C13—H13	119.8	N1—C26—C23	107.3 (2)
C11—C12—C13	121.4 (5)	C25—C26—C23	110.5 (2)
C11—C12—H12	119.3	C24—C26—C23	112.1 (2)
C13—C12—H12	119.3		

### Hydrogen-bond geometry (Å, º)

**Table:**

*D*—H···*A*	*D*—H	H···*A*	*D*···*A*	*D*—H···*A*
N1—H1*C*···O8^i^	0.99	1.85	2.800 (4)	160
N1—H1*B*···O5^ii^	0.91	2.04	2.935 (3)	166
N1—H1*A*···O6^iii^	0.97	1.88	2.779 (3)	153
O5—H5···O7^iii^	0.97	1.76	2.714 (3)	170
O6—H6···O1	0.91	1.81	2.662 (3)	154
O7—H7*A*···O8^iii^	0.92	1.77	2.686 (3)	173
O8—H8*B*···O2	0.92	1.76	2.617 (3)	1523
O8—H8*A*···O1^iv^	0.91	1.76	2.667 (3)	173
C24—H24*A*···O1	0.99	2.55	3.410 (4)	146
C25—H25*A*···O2^iii^	0.99	2.44	3.326 (4)	149

Symmetry codes: (i) −*x*+1, −*y*+1, −*z*+1; (ii) −*x*+1, *y*+1/2, −*z*+1/2; (iii) *x*, −*y*+3/2, *z*−1/2; (iv) *x*, −*y*+1/2, *z*+1/2.
